# The conditional deletion of steroidogenic factor 1 (*Nr5a1*) in *Sox9-Cre* mice compromises testis differentiation

**DOI:** 10.1038/s41598-021-84095-y

**Published:** 2021-02-24

**Authors:** Yayoi Ikeda, Ayako Tagami, Mamiko Maekawa, Akiko Nagai

**Affiliations:** Department of Anatomy, Aichi Gakuin University School of Dentistry, 1-100 Kusumoto-Cho, Chikusa-ku, Nagoya, Aichi 464-8650 Japan

**Keywords:** Differentiation, Endocrinology

## Abstract

Steroidogenic factor 1 (NR5A1) is essential for gonadal development. To study the importance of NR5A1 during early gonadal sex differentiation, we generated *Sox9-Cre*-*Nr5a1* conditional knockout (cKO) mice: *Sox9-Cre;Nr5a1*^*flox/flox*^ and *Sox9-Cre;Nr5a1*^*flox/−*^ mice. Double-immunostaining for NR5A1 and AMH revealed silenced NR5A1 in Sertoli cells and reduced AMH^+^ cells in the gonads of XY *Sox9-Cre*-*Nr5a1* cKO mice between embryonic days 12.5 (E12.5) and E14.5. Double-immunostaining for SOX9 and FOXL2 further indicated an early block in Sertoli cells and ectopic granulosa cell differentiation. The number of cells expressing the Leydig cell marker 3βHSD obviously reduced in the gonads of XY *Sox9-Cre;Nr5a1*^*flox/−*^ but not *Sox9-Cre;Nr5a1*^*flox/flox*^ mice at E15.5. The presence of STRA8^+^ cells indicated that germ cells entered meiosis in the gonads of XY *Sox9-Cre*-*Nr5a1* cKO mice. The results of qRT-PCR revealed remarkably reduced and elevated levels of testis and ovary markers, respectively, in the gonads of XY *Sox9-Cre*-*Nr5a1* cKO mice at E12.5‒E13.5. These data suggested that the loss of *Nr5a1* abrogates the testicular pathway and induces the ectopic ovarian pathway, resulting in postnatal partial/complete male-to-female gonadal sex reversal. Our findings provide evidence for the critical role of NR5A1 in murine gonadal sex determination in vivo.

## Introduction

In mammals, bi-potential undifferentiated gonads, which develop from the gonadal primordium in the urogenital ridge, differentiate into either testes or ovaries^1,2^. Testis differentiation is primarily determined by the transient expression of sex-determining region Y (*Sry*), a Y chromosome-linked gene expressed at E10.5–E12.5 in mice^3–6^. The expression of sex-determining region-box 9 (*Sox9*) is upregulated in male gonads by SRY, specifically in Sertoli-lineage cells at E11.5. Consequently, the supporting Sertoli cell population concomitantly differentiates with the formation of testis cords that enclose both Sertoli and germ cells. Subsequently, steroidogenic Leydig cells differentiate in the interstitium between the cords around E12.5.

The nuclear hormone receptor, steroidogenic factor 1 (NR5A1, also known as SF-1 or Ad4BP) was primarily identified as a key regulator of steroid hormone biosynthesis in adult animals^7^. Tissues involved in the development and function of the endocrine/reproductive system, including the adrenal and pituitary glands, the ventromedial hypothalamus, ovaries, and testes, express *Nr5a1*, and notably, the murine urogenital ridge in both sexes also expresses *Nr5a1* from E9.5^8^. However, around the time of testicular morphogenesis at E12.5, *Nr5a1* expression increases in the testes and decreases in the ovaries^8,9^. In vitro studies have found that SRY up-regulates *Sox9* expression via interaction with NR5A1 in somatic cells to induce pre-Sertoli, and ultimately Sertoli cells, suggesting that NR5A1 plays a critical role in early testis differentiation^4,5,10,11^.

Gonadal development is transiently delayed in heterozygous *Nr5a1* mutant mice, but they do not undergo XY sex reversal^12,13^. The gonads of homozygous *Nr5a1* null mutant (global *Nr5a1*^*−/−*^) mice undergo apoptosis-mediated degeneration around E11.5, which is immediately before testis morphogenesis^14^. We generated conditional, gonad-specific *Nr5a1* knockout mice using the *Cre-loxP* system to circumvent gonadal agenesis and follow the development of global *Nr5a1*^*−/−*^ gonads in vivo. We previously generated Anti-Müllerian hormone receptor type 2 (*Amhr2*)*-Cre Nr5a1* conditional knockout (*Amhr2-Cre-Nr5a1* cKO) mice, in which *Nr5a1* was specifically ablated in foetal Leydig cells. The XY *Amhr2-Cre-Nr5a1* cKO mice showed hypoplastic testes with histological abnormalities and internal female structures within the abdominal cavity, suggesting that NR5A1 functions in the development of male reproductive organs^15^. Since *Nr5a1* is also deleted in Sertoli cells in postnatal *Amhr2-Cre-Nr5a1* cKO mice, analyses of the expression of Sertoli cell markers revealed that NR5A1 plays an important role in Sertoli cell maturation and spermatogenesis during postnatal testis development^16^. Anti-Müllerian hormone (*Amh,* also known as Müllerian inhibiting substance*, Mis*)*-Cre*-mediated *Nr5a1* cKO mice have also been generated^17^. The gonads of XY *Amh-Cre-Nr5a1* cKO mice are strikingly hypoplastic, and the loss of Sertoli and germ cells along with dysgenic testis cords from E15.5 suggested a role for NR5A1 in Sertoli cell survival and proliferation during embryonic testis differentiation after sex determination. However, despite *Nr5a1* upregulation in the testis as early as E12.5, *Nr5a1* was inactivated at E14.5 in XY *Amh-Cre-Nr5a1* cKO mouse gonads. Thus, the role of NR5A1 in testis differentiation at E12.5–E14.5 has remained unclear.

To study the role of NR5A1 during early testis differentiation, we generated a different *Nr5a1* cKO using the *Sox9-Cre* transgene, which is active in the gonads, osteo-chondrogenic tissues, intestine, spinal cord, and pancreas, during mouse embryogenesis^18^. Because the expression of *Sox9* is specifically elevated in Sertoli-lineage cells from the beginning of testis differentiation^4,5,10^, *Sox9-Cre* should induce a deletion of the *Nr5a1* allele before *Amh-Cre*, in which the Cre recombinase is expressed at E14.5^17^. Here, we generated genotypically different *Sox9-Cre;Nr5a1*^*flox/flox*^ cKO (*Sox9-Cre;Nr5a1f.*^*/f*^) and *Sox9-Cre;Nr5a1*^*flox/−*^ cKO (*Sox9-Cre;Nr5a1f.*^*/−*^) mice that respectively exhibited postnatal partial and complete XY sex reversal. We analysed the expression of cell type-specific gonadal markers between E12.5 and E15.5 to determine the onset and progression of sex reversal in XY *Sox9-Cre-Nr5a1* cKO mice.

## Results

### Generation of *Sox9-Cre Nr5a1* cKO mice

Using *Sox9-Cre*, we generated two genotypes of homozygous *Nr5a1* cKO mice, *Sox9-Cre;Nr5a1f.*^*/f*^ and *Sox9-Cre;Nr5a1f.*^*/−*^. Immunohistochemistry (IHC) findings confirmed that Cre was expressed by Sertoli cells in XY *Sox9-Cre*, but not in XY *Nr5a1f.*^*/−*^ mice at E12.5–E15.5 (Supplementary Fig. 1). Fewer Cre^+^ cells were found in XY mice of both *Sox9-Cre-Nr5a1* cKO genotypes than in XY *Sox9-Cre;Nr5a1f.*^*/*+^ control mice at E13.5‒E15.5. Since the effects of *Nr5a1* are dose-dependent in developing testes^13^, we also examined *Nr5a1*^+*/−*^ heterozygous mice. *Nr5a1f.*^*/f*^, *Nr5a1f.*^*/*+^, and *Sox9-Cre;Nr5a1f.*^*/*+^ littermates were assessed as controls. Because the IHC findings of NR5A1 and markers of Sertoli, Leydig, and germ cells did not differ among the three control groups (Supplementary Fig. 2), these groups were combined in the subsequent analyses.

### Distinct postnatal phenotypes of genetically different *Sox9-Cre-Nr5a1* cKO mice

**Figure 1:**
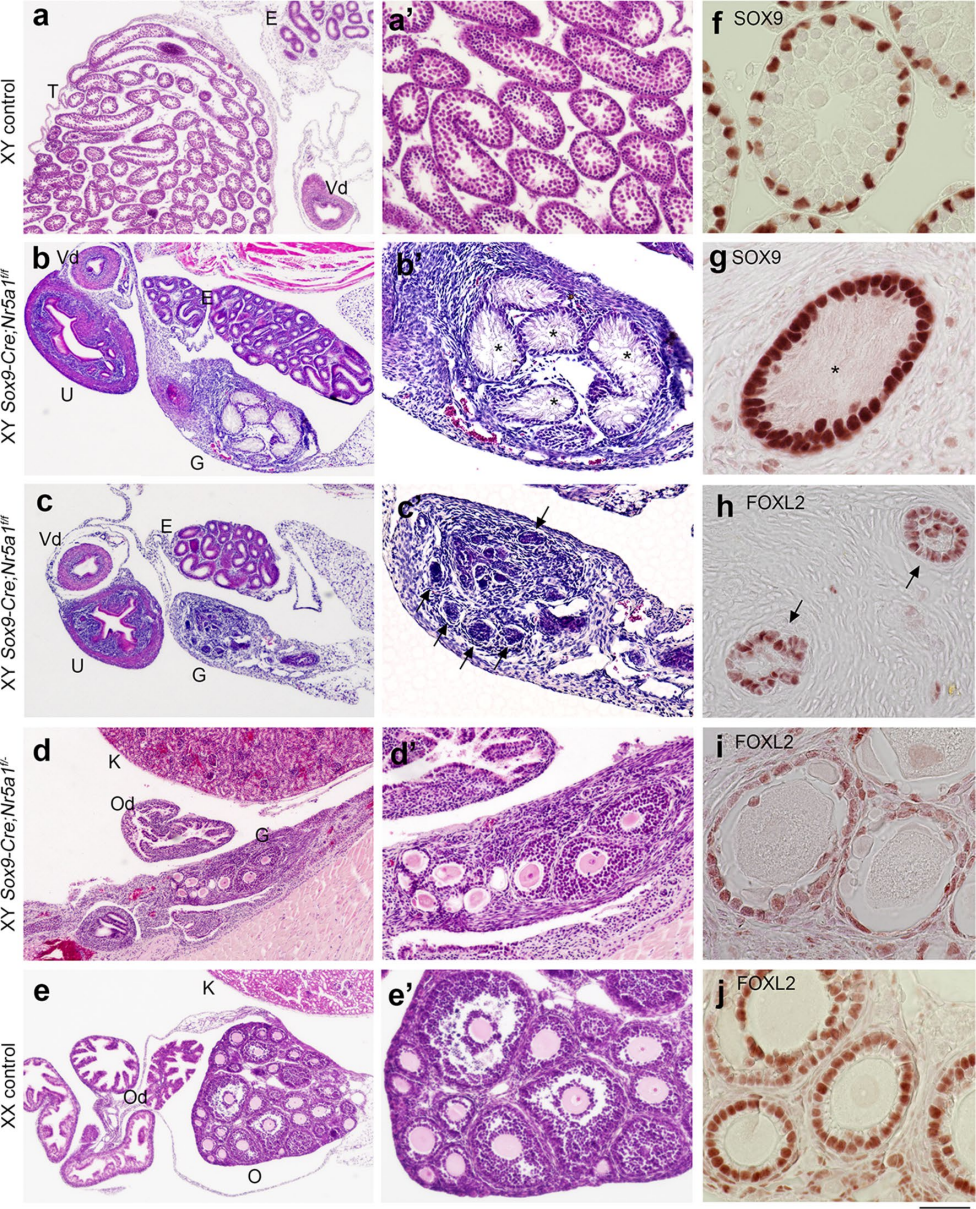
Gonadal phenotypes of postnatal XY *Sox9-Cre*-*Nr5a1* cKO mice. (**a**–**e**) Haematoxylin and eosin (HE) staining. (**a′**–**e′)** Higher magnification views. Representative photomicrographs of XY control (**a**), XY *Sox9-Cre;Nr5a1f.*^*/f*^ (**b**,**c**), XY *Sox9-Cre;Nr5a1f.*^*/−*^ (**d**), and XX control (**e**) gonads from 3 week-old animals. (**b**) and (**c**) are from serial sections of the same gonad. (**f**–**j**) IHC for SOX9 (**f**,**g**) or FOXL2 (**h**–**j**). Asterisks in (**b′**) and (**g**) and arrows in (**c′**) and (**h**) indicate seminiferous tubule-like and ovarian follicle-like structures, respectively. *E* epididymis, *G* gonad, *K* kidney, *O* ovary, *Od* oviduct, *T* testis, *U* uterus, *Vd* vas deferens. Scale bar, 250 µm in (**a**–**e**), 100 µm in (**a′–e′**), 25 µm in (**f–j**).

Gross observation of XY *Sox9-Cre;Nr5a1f.*^*/f*^ mice revealed ambiguous external genitalia and two internal reproductive tracts with very small gonads, located at the level of the bladder two weeks after birth (Supplementary Fig. 3). On the other hand, external genitalia were indistinguishable between XY *Sox9-Cre;Nr5a1f.*^*/−*^ and XX control female mice (Supplementary Fig. 4). An internal uterus was identified, but gross observation did not reveal any gonads. We then histologically analysed the reproductive tissues. Two reproductive tracts were defined as a uterus and vas deferens in XY *Sox9-Cre;Nr5a1f.*^*/f*^ mice (Fig. 1b,c), and their gonads were markedly smaller than those of XY control mice (Fig. 1a). The gonads (Fig. 1b) comprise a large interstitial area comprising connective tissue and a few irregularly shaped tubule-like structures (asterisks in Fig. 1b′) that were similar to seminiferous tubules (Fig. 1a′) in size and enclosed Sertoli-like cells but not male gametes. Other sections of the same gonad (Fig. 1c) contained a few small vesicular structures (arrows in Fig. 1c′). We also used IHC to detect SOX9 and Forkhead box protein L2 (FOXL2), markers of Sertoli cells (Fig. 1f) and granulosa cells (Fig. 1j) in the testes and the ovaries, respectively. Tubule-like and vesicular structures contained SOX9^+^ (Fig. 1g) and FOXL2^+^ cells (Fig. 1h), respectively, indicating that the gonads of XY *Sox9-Cre;Nr5a1f.*^*/f*^ mice were “ovotestes”, with a testicular portion comprising seminiferous-like tubules and an ovarian portion with follicle-like structures, and both portions were atypical. We examined six gonads from XY *Sox9-Cre;Nr5a1f.*^*/f*^ mice and did not identify any apparent variability in histological and IHC results among them. Histological staining showed that gonads of XY *Sox9-Cre;Nr5a1f.*^*/−*^ mice were < 1 mm in length and were located below the kidneys, where ovaries are usually positioned (Fig. 1d,e). All 10 histologically stained gonads from XY *Sox9-Cre;Nr5a1f.*^*/−*^ mice were ovaries with primary and secondary follicles (Fig. 1d′,e′) comprising FOXL2^+^ granulosa cells (Fig. 1i), similar to those in XX control ovaries (Fig. 1j).

### Reduced numbers of Sertoli cells in the gonads of XY *Sox9-Cre-Nr5a1* cKO mice

**Figure 2:**
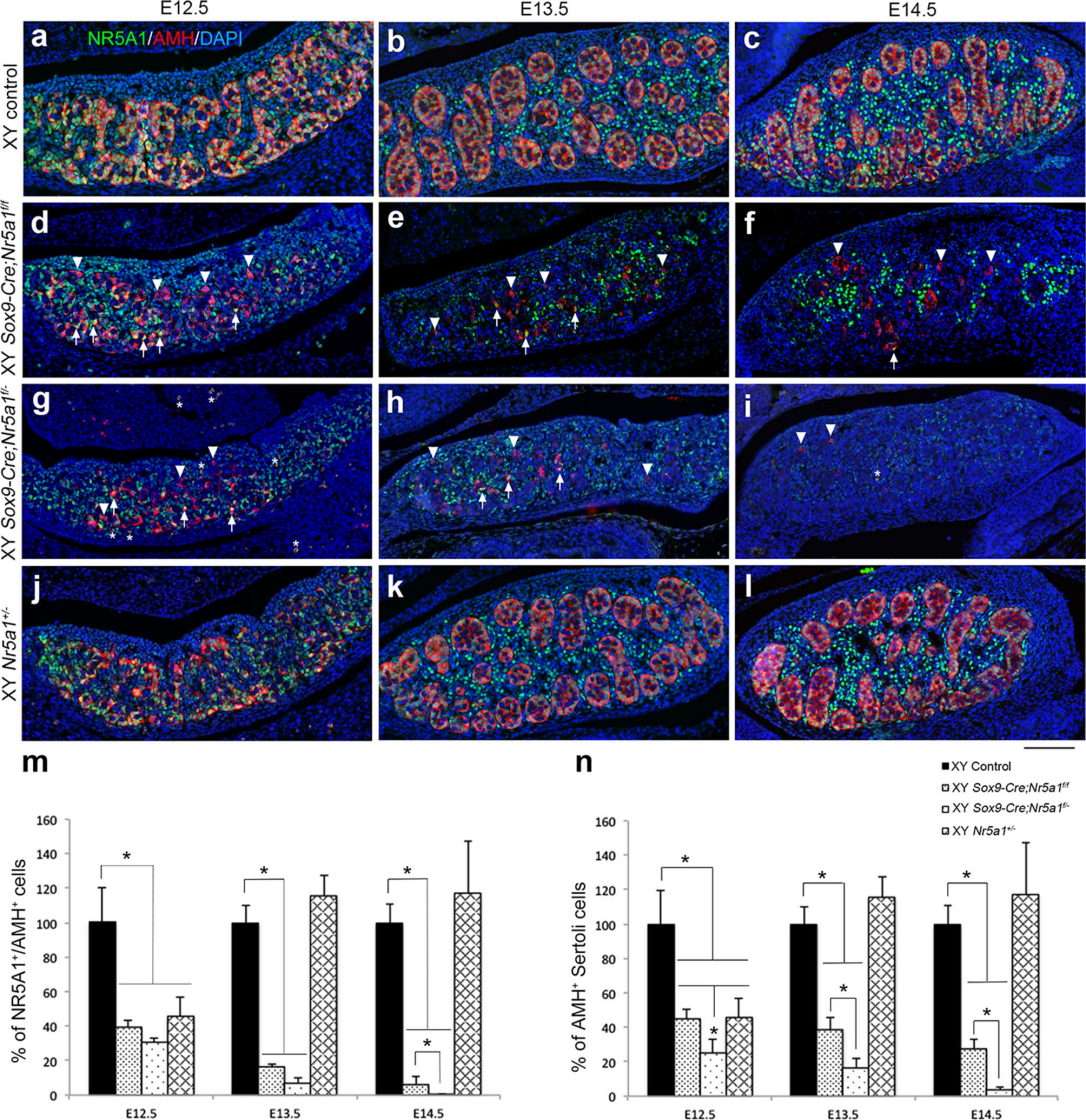
*Nr5a1* inactivation in Sertoli cells during early embryonic development. Double IHC for NR5A1 (green) and AMH (red; Sertoli cell marker) in gonads of XY control (**a**–**c**), XY *Sox9-Cre;Nr5a1f.*^*/f*^ (**d**–**f**), XY *Sox9-Cre;Nr5a1f.*^*/−*^ (**g**–**i**), and XY *Nr5a1*^+*/−*^ (**j**–**l**) mice at E12.5, E13.5, and E14.5 (left, middle, and right panels, respectively). Arrows and arrowheads indicate representative NR5A1^+^/AMH^+^ and NR5A1^−^/AMH^+^ cells, respectively. White asterisks in (**g**) and (**i**) indicate nonspecific autofluorescence. Ventral, anterior, and posterior sides of gonads are upwards, right, and left, respectively. Nuclei are stained blue with DAPI. Scale bar, 100 µm. Ratios (%) of NR5A1^+^/AMH^+^ (**m**) and AMH^+^ (**n**) cells. (Daily values for XY control were set at 100%). Data are shown as means ± SEM (n = 3). *P < 0.05 vs. XY control.

To examine the timing of *Nr5a1* inactivation in Sertoli cells, gonadal sections from XY control, *Sox9-Cre;Nr5a1f.*^*/f*^*, Sox9-Cre;Nr5a1f.*^*/−*^, and *Nr5a1*^+*/−*^ mice were double-stained for NR5A1 and AMH, a marker of Sertoli cells (Fig. 2). The IHC findings revealed that AMH^+^ cells co-labelled with NR5A1 (NR5A1^+^/AMH^+^) in XY control and XY *Nr5a1*^+*/−*^ gonads (Fig. 2a–c, j–l), and with NR5A1^+^/AMH^+^ (arrows in Fig. 2d–h) and NR5A1^−^/AMH^+^ cells (arrowheads in Fig. 2d–i) in the gonads of both XY *Sox9-Cre-Nr5a1* cKO genotypes.

The numbers of NR5A1^+^/AMH^+^ cells in both genotypes of XY *Sox9-Cre-Nr5a1* cKO mice were < 50% of that in XY controls at E12.5, and < 10% and 1% in XY *Sox9-Cre;Nr5a1f.*^*/f*^ and *Sox9-Cre;Nr5a1f.*^*/−*^ gonads, respectively, at E14.5 (Fig. 2m). We also counted AMH^+^ cells, including NR5A1^+^/AMH^+^ and NR5A1^−^/AMH^+^ cells, and found fewer AMH^+^ Sertoli cells in the XY *Sox9-Cre-Nr5a1* cKO mice; significantly fewer AMH^+^ cells were found in XY *Sox9-Cre;Nr5a1f.*^*/−*^ than in XY *Sox9-Cre;Nr5a1f.*^*/f*^ gonads at E12.5‒E14.5 (Fig. 2n). The numbers of SOX9^+^ Sertoli cells were similarly low in XY *Sox9-Cre-Nr5a1* cKO gonads at E12.5-E14.5 (Supplementary Fig. 5). The number of AMH^+^ cells in XY *Nr5a1*^+*/−*^ gonads was notably ~ 50% of that in XY controls at E12.5 but returned to XY control levels by E13.5 (Fig. 2n).

### Aberrant sexual differentiation of supporting cells in XY *Sox9-Cre-Nr5a1* cKO gonads

**Figure 3:**
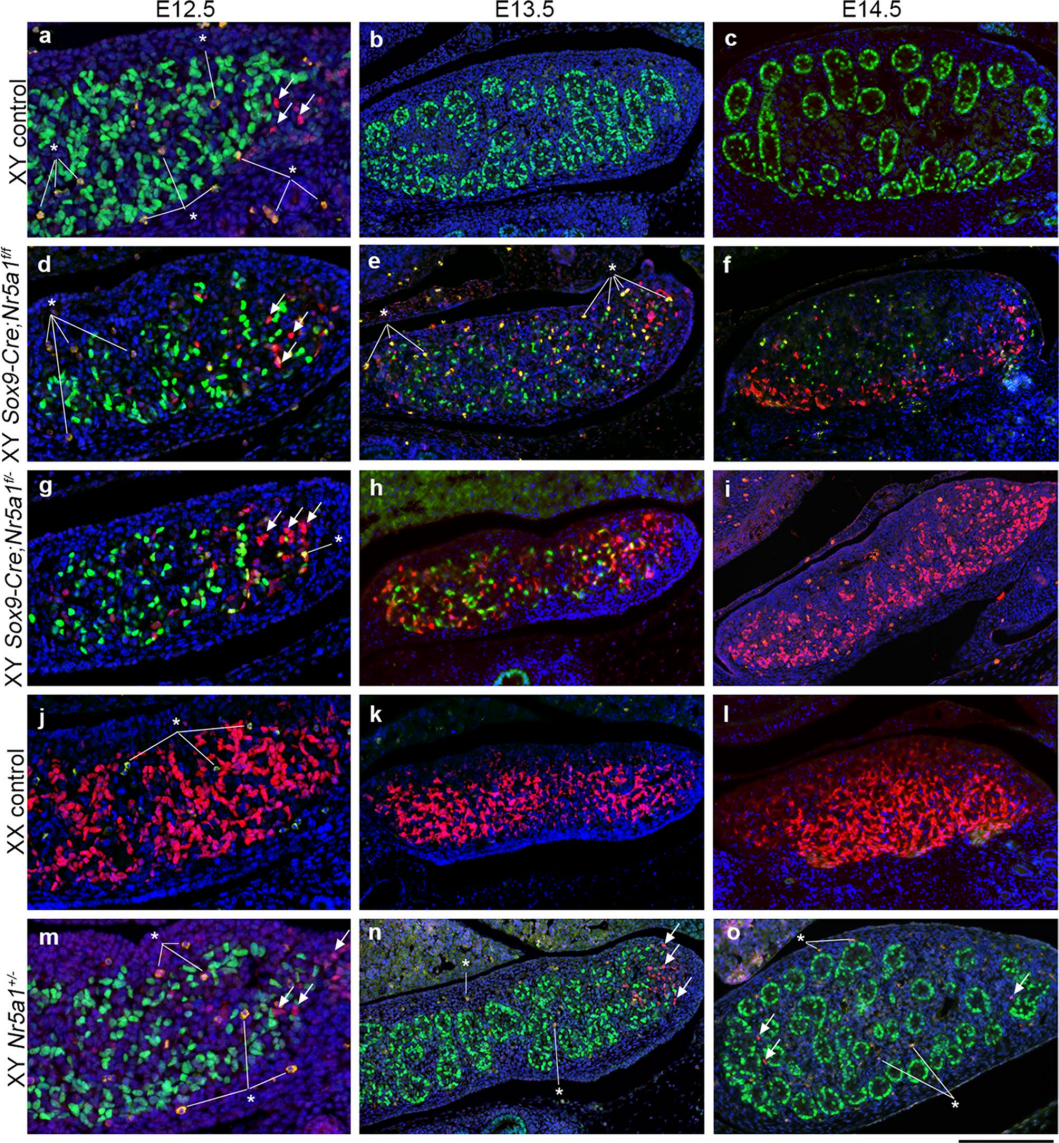
Sexual differentiation of supporting cells in XY *Sox9-Cre*-*Nr5a1* cKO gonads. Double IHC for Sertoli cell marker SOX9 (green) and granulosa cell marker FOXL2 (red) in gonads of XY control (**a**–**c**), XY *Sox9-Cre;Nr5a1f.*^*/f*^ (**d**–**f**), XY *Sox9-Cre;Nr5a1f.*^*/−*^ (**g**–**i**), XX control (**j**–**l**), and XY *Nr5a1*^+*/−*^ (**m**–**o**) mice at E12.5, E13.5, and E14.5 (left, middle, and right panels, respectively). Ventral, anterior, and posterior sides of gonad are upwards, right, and left, respectively. White arrows indicate representative FOXL2^+^ cells in XY gonads. White asterisks indicate nonspecific autofluorescence. Nuclei are stained blue with DAPI. Scale bar, 50 µm in (**a**,**d**,**g**,**j**,**m**), 100 μm in (**b**,**c**,**e**,**f**,**h**,**i**,**k**,**l**,**n**,**o**).

We double-stained SOX9 and FOXL2 to assess whether the early loss of *Nr5a1* affects the sexual differentiation of supporting cells in XY gonads (Fig. 3). We detected SOX9^+^ cells in XY but not in XX control gonads at E12.5–E14.5 (Fig. 3a–c, j–l), but their numbers were reduced in XY *Sox9-Cre-Nr5a1* cKO gonads at E12.5–E14.5 (Fig. 3d–i, Supplementary Fig. 5). On the other hand, FOXL2^+^ cells were abundant in XX control gonads at E12.5‒E14.5 (Fig. 3j–l), whereas FOXL2^+^ cells in XY gonads at E12.5 were barely detectable at E13.5–E14.5 (Fig. 3a–c). The number of FOXL2^+^ cells in gonads from the two XY *Sox9-Cre-Nr5a1* cKO models at E12.5 gradually increased at E13.5–E14.5 (Fig. 3d–i). A few FOXL2^+^ cells were also detected in XY *Nr5a1*^+*/−*^ gonads at E12.5–E14.5 (Fig. 3m–o).

### Differential perturbation of Leydig cells in XY *Sox9-Cre-Nr5a1* cKO gonads

**Figure 4:**
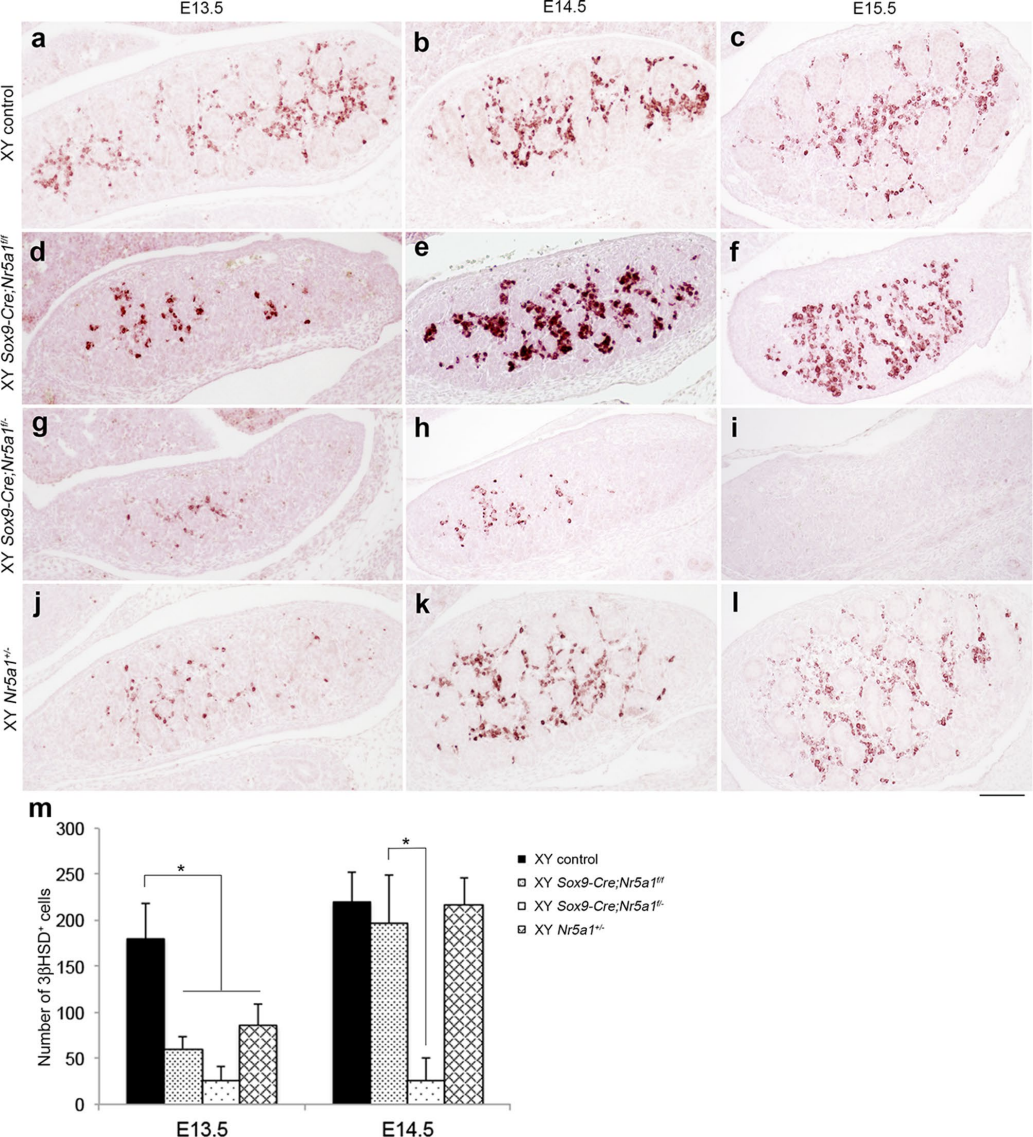
Leydig cell differentiation in XY *Sox9-Cre*-*Nr5a1* cKO gonads. IHC for Leydig cell marker, 3βHSD in gonads of XY control (**a**–**c**), XY *Sox9-Cre;Nr5a1f.*^*/f*^ (**d**–**f**), XY *Sox9-Cre;Nr5a1f.*^*/−*^ (**g**–**i**), and XY *Nr5a1*^+*/−*^ (**j**–**l**) mice at E13.5, E14.5, and E15.5 (left, middle, and right panels, respectively). Ventral, anterior, and posterior sides of gonad are upwards, right, and left, respectively. Scale bar, 100 μm. (**m**) Numbers of 3βHSD^+^ cells in gonadal section determined by IHC. Data are shown as means ± SEM (n = 3). *P < 0.05.

We assessed the expression of 3 beta-hydroxysteroid dehydrogenase (3βHSD) using IHC to determine Leydig cell differentiation (Fig. 4). The expression of 3βHSD was detected in the interstitial region of XY control gonads at E13.5–E15.5 (Fig. 4a–c). 3βHSD^+^ cells were fewer in number in the gonads from the two XY *Sox9-Cre-Nr5a1* cKO models and from XY *Nr5a1*^+*/−*^ mice compared with those in the gonads of XY control animals at E13.5 (Fig. 4d,g,j,m). The number of 3βHSD^+^ cells in gonads from XY *Sox9-Cre;Nr5a1f.*^*/f*^ and *Nr5a1*^+*/−*^ animals returned to levels that were equivalent to those in XY control gonads by E14.5 (Fig. 4e,f,k,l,m). However, the number of 3βHSD^+^ cells remained low in XY *Sox9-Cre;Nr5a1f.*^*/−*^ gonads at E14.5, and the cells were undetectable at E15.5 (Fig. 4h,i,m).

### Persistence of germ cells and their meiotic entry in XY *Sox9-Cre-Nr5a1* cKO gonads

**Figure 5:**
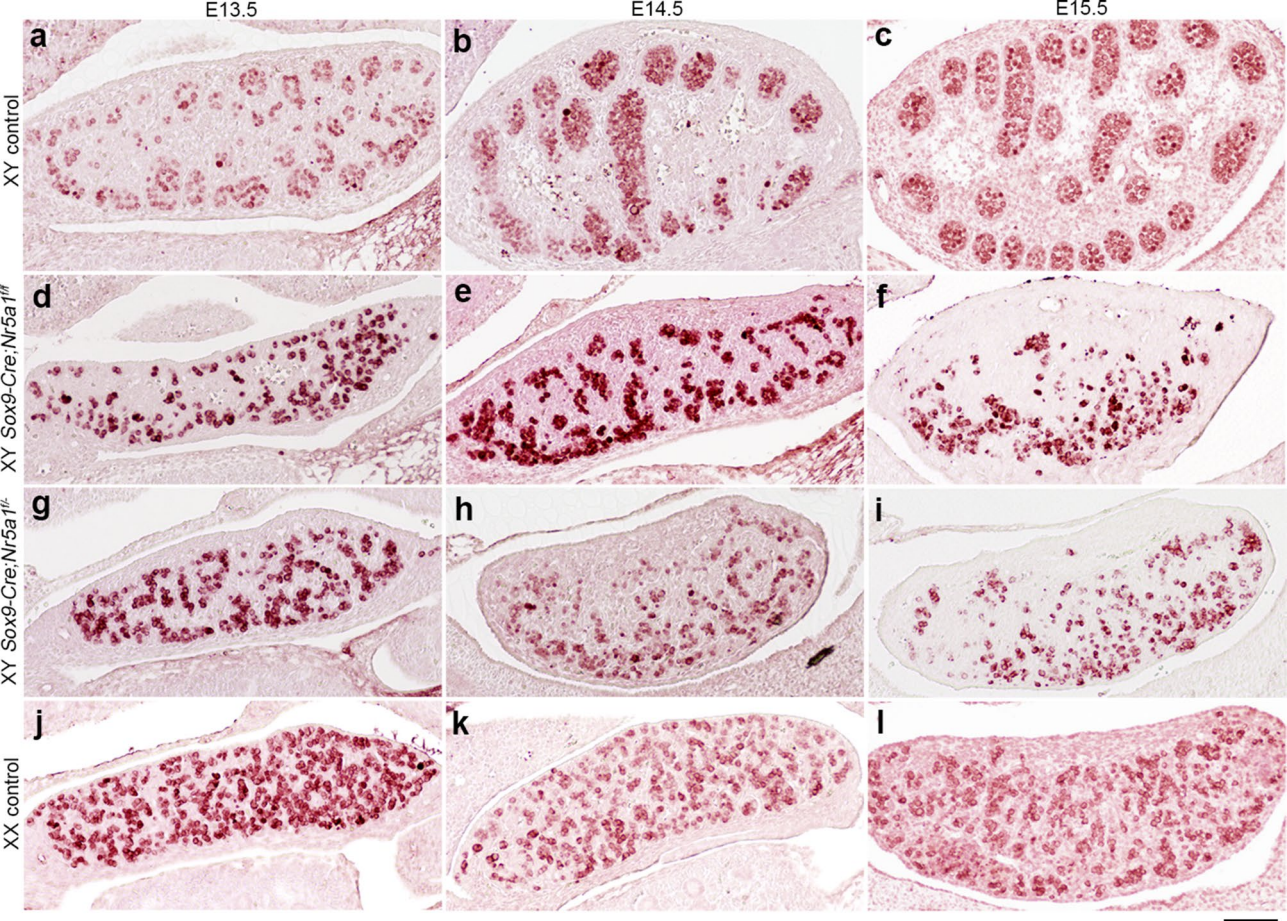
Distribution of germ cells in XY *Sox9-Cre*-*Nr5a1* cKO gonads. IHC for DDX4, a germ cell marker, in gonads of XY control (**a**–**c**), XY *Sox9-Cre;Nr5a1f.*^*/f*^ (**d**–**f**), XY *Sox9-Cre;Nr5a1f.*^*/−*^ (**g**–**i**), and XX control (**j**–**l**) mice at E13.5, E14.5, and E15.5 (left, middle, and right panels, respectively). Ventral, anterior, and posterior sides of gonad are upwards, right, and left, respectively. Scale bar, 100 μm.

**Figure 6:**
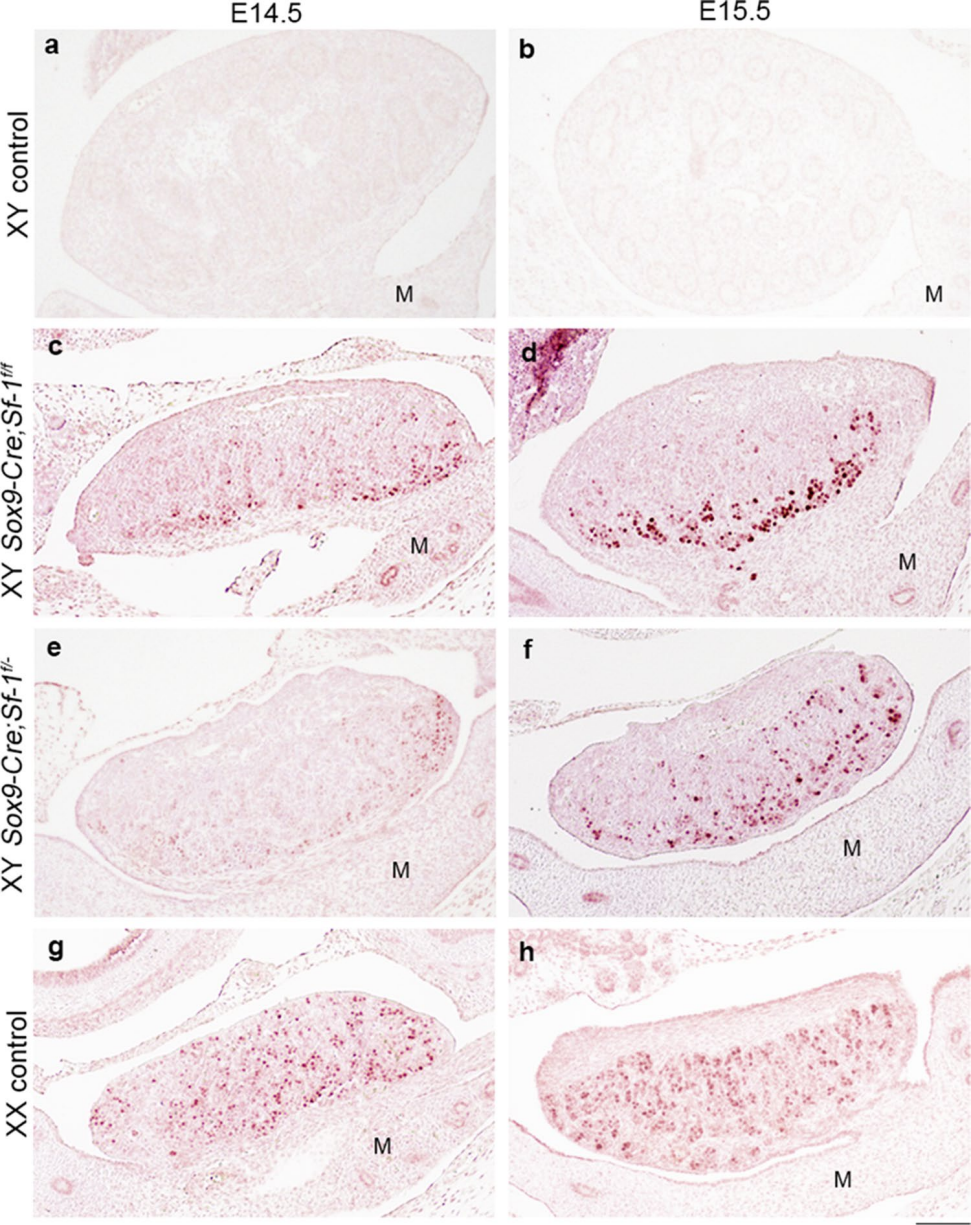
Presence of meiotic germ cells in XY *Sox9-Cre*-*Nr5a1* cKO gonads. IHC for meiotic germ cell marker, STRA8, in gonads of XY control (**a**,**b**), *Sox9-Cre;Nr5a1f.*^*/f*^ (**c**,**d**), XY *Sox9-Cre;Nr5a1f.*^*/−*^ (**e**,**f**), and XX control (**g**,**h**) mice at E14.5 and E15.5 (left and right panels, respectively). Ventral, anterior, and posterior sides of gonad are upwards, right, and left, respectively. M, mesonephros. Scale bar, 100 μm.

Mouse primordial germ cells migrate into the urogenital ridge around E10.5, proliferate until E13.5, and then become mitotically arrested in male, or enter meiosis in female gonads. Here, we performed IHC for DEAD [Asp-Glu-Ala-Asp] box polypeptide 4 (DDX4, also known as VASA), a marker of germ cells (Fig. 5). DDX4^+^ cells were enclosed within the testis cord in XY control gonads (Fig. 5a–c) and were evenly distributed within the gonads of XX controls (Fig. 5j–l). The distribution of DDX4^+^ cells in XY *Sox9-Cre-Nr5a1* cKO gonads appeared irregular (Fig. 5d–i). To determine germ cell differentiation, we examined the expression of stimulated by retinoic acid gene 8 (STRA8), a marker of meiotic germ cells, also via IHC (Fig. 6). We found STRA8^+^ cells in XX (Fig. 6g,h) but not in XY (Fig. 6a,b) control gonads at E14.5 and E15.5. On the other hand, STRA8^+^ cells were detected in XY *Sox9-Cre-Nr5a1* cKO gonads (Fig. 6c–f), but appeared less abundant than those in XX controls and tended to localise to the pole and dorsal sides.

### Impairment of testis cord organisation in XY *Sox9-Cre-Nr5a1* cKO gonads

**Figure 7:**
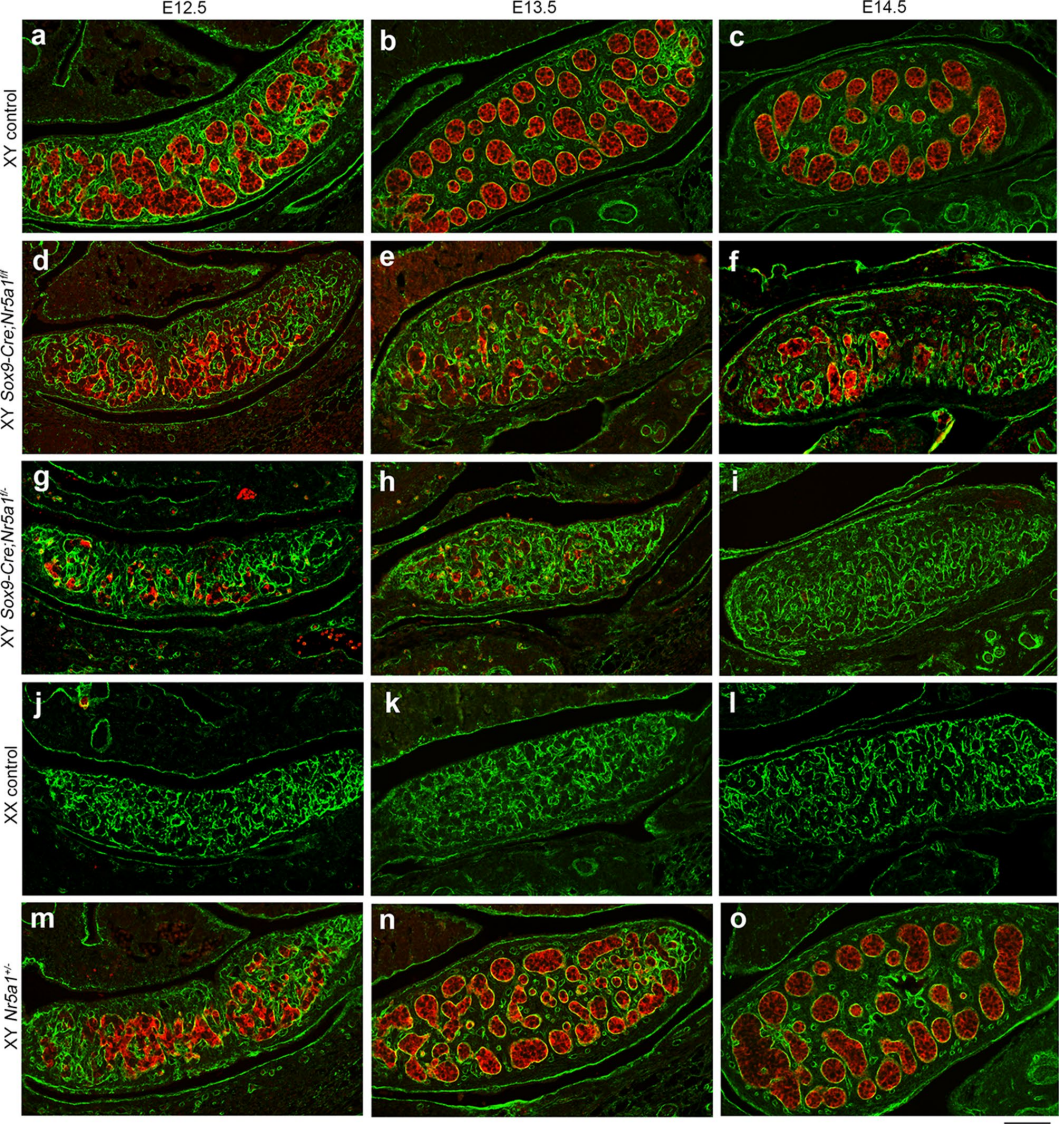
Disrupted testis cord formation in XY *Sox9-Cre*-*Nr5a1* cKO gonads. Double IHC for laminin (green) and AMH (red) in gonads of XY control (**a**–**c**), XY *Sox9-Cre;Nr5a1f.*^*/f*^ (**d**–**f**), XY *Sox9-Cre;Nr5a1f.*^*/−*^ (**g**–**i**), XX control (**j**–**l**), and XY *Nr5a1*^+*/−*^ (**m**–**o**) mice at E12.5, E13.5, and E14.5 (left, middle, and right panels, respectively). Ventral, anterior, and posterior sides of gonad are upwards, right, and left, respectively. Scale bar, 100 μm.

Foetal testes are morphologically characterised by the formation of testis cords that enclose Sertoli cells. Because the basement membrane of testis cords can be outlined using laminin immunostaining, we investigated testis cord formation by double staining for laminin and AMH (Fig. 7). Normal testicular cords enclosing AMH^+^ cells were detected in XY (Fig. 7a–c) but not in XX (Fig. 7j–l) control gonads at E12.5–E14.5, as expected. The testis cords in XY *Sox9-Cre;Nr5a1f.*^*/f*^ gonads were morphologically irregular and narrower than those in the XY controls at E12.5, and were only partially detected at E14.5 (Fig. 7d–f). The cords in XY *Sox9-Cre;Nr5a1f.*^*/−*^ mice appeared more disorganised than those in XY *Sox9-Cre;Nr5a1f.*^*/f*^ gonads at E12.5 and E13.5, and the structure outlined by laminin resembled that of control XX ovaries at E14.5 (Fig. 7g–i). The testis cords in XY *Nr5a1*^+*/−*^ mice also appeared irregular at E12.5 and E13.5, but were indistinguishable from those of XY controls at E14.5 (Fig. 7m–o).

### Expression levels of *Nr5a1* and gonadal genes

**Figure 8:**
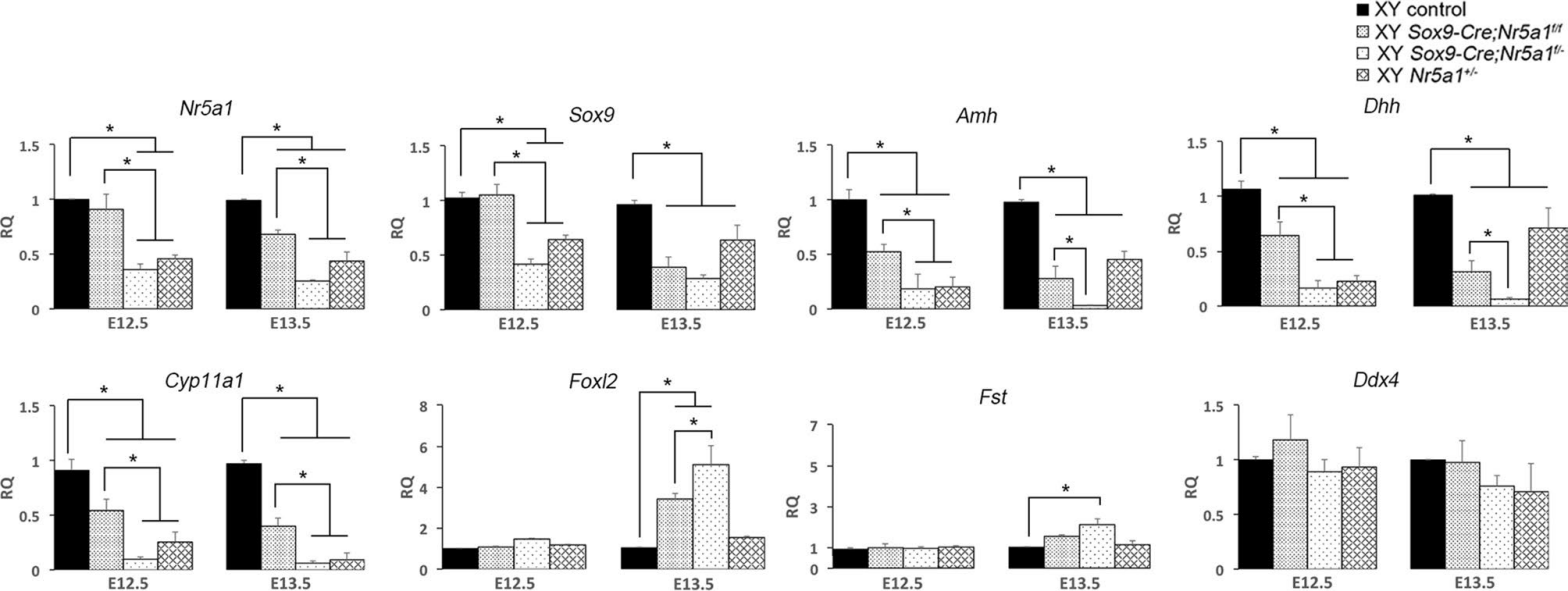
Gene expression in XY *Sox9-Cre*-*Nr5a1* cKO gonads. Expression of *Nr5a1*, *Sox9*, *Amh,* and *Dhh*, *Cyp11a1*, *Foxl2*, *Fst*, and *Ddx4* at E12.5 and E13.5 was analysed using qRT-PCR. Values for XY control, XY *Sox9-Cre;Nr5a1f.*^*/f*^, XY *Sox9-Cre;Nr5a1f.*^*/−*^, and XY *Nr5a1*^+*/−*^ gonads are relative to the expression of 18S ribosomal RNA, and normalised to that of XY control mRNA (set to 1). Data are shown as means ± SEM (n = 3). *P < 0.05.

We compared the expression levels of genes involved in gonadal development in XY control, *Sox9-Cre-Nr5a1* cKO, and *Nr5a1*^+*/−*^ gonads using quantitative real-time PCR (qRT-PCR) (Fig. 8). Levels of *Nr5a1* in XY *Sox9-Cre;Nr5a1f.*^*/f*^ gonads were comparable, reaching ~ 60% of those in XY control gonads at E12.5 and E13.5, respectively, whereas those in XY *Sox9-Cre;Nr5a1f.*^*/−*^ gonads were < 50% of those in XY controls between E12.5 and E13.5. We also analysed the expression of the testis markers, *Sox9, Amh,* desert hedgehog *(Dhh)*, and cytochrome P450 family 11 subfamily A member 1 *(Cyp11a1,* also known as *P450scc)*. Levels of *Sox9* in XY *Sox9-Cre;Nr5a1f.*^*/f*^ gonads were comparable to those in XY controls at E12.5, but were reduced to < 50% at E13.5; those in XY *Sox9-Cre;Nr5a1f.*^*/−*^ gonads were < 50% at E12.5‒E13.5. Moreover, levels of *Amh, Dhh*, and *Cyp11a1* expression were significantly lower in both XY *Sox9-Cre-Nr5a1* cKO models than in XY controls, and significantly lower in XY *Sox9-Cre;Nr5a1f.*^*/−*^ than in XY *Sox9-Cre;Nr5a1f.*^*/f*^ gonads at E12.5–E13.5. The levels of testis markers were also significantly lower in XY *Nr5a1*^+*/−*^ than in XY control gonads at E12.5–E13.5. The ovary markers *Foxl2* and follistatin *(Fst)* were similarly expressed in all groups at E12.5, but tended to be elevated in XY *Sox9-Cre-Nr5a1* cKO compared with levels in XY control gonads at E13.5. The expression of the germ cell marker, *Ddx4*, did not significantly differ among the groups.

## Discussion

XY *Amh-Cre*-*Nr5a1* cKO mice lacking *Nr5a1* in Sertoli cells at E14.5 showed a reduction of Sertoli cell numbers and a loss of proliferating Sertoli cells at E15.5–E18.5, suggesting a role for NR5A1 in the regulation of Sertoli cell proliferation^17^. We used the *Sox9-Cre* transgene to investigate the importance of NR5A1 in the early stages of testes differentiation in vivo, because *Sox9* is specifically elevated in Sertoli cells from the beginning of testis differentiation. The phenotypes of postnatal XY *Sox9-Cre-Nr5a1* cKO gonads with partial and complete male-to-female sex reversal were obviously more severe than those of *Amh-Cre-Nr5a1* cKO mice. The results of IHC for AMH and SOX9 indicated that both XY *Sox9-Cre-Nr5a1* cKO models had conspicuously less Sertoli cells at E12.5–E14.5. The spatial and temporal IHC findings of gonadal protein markers during the early phase of testis morphogenesis revealed novel and important information about the cellular consequences of *Nr5a1* loss.

*Sox9-Cre* should have inactivated *Nr5a1* along with elevated *Sox9* expression in Sertoli cells. However, undifferentiated somatic cells in indifferent gonads of both sexes express low levels of *Sox9* at E11.5‒E12.5^10^. Furthermore, all mesenchymal cells including Sertoli cells and Leydig cells are β-gal-positive in *Sox9-Cre;R26R* embryos at E17.5^18^. Somatic cells in indifferent gonads of both sexes express *Nr5a1* as early as E9.5^8^. Therefore, if the *Sox9* levels in indifferent gonads are sufficient to cause Cre expression in *Sox9-Cre* mice, *Nr5a1* ablation may occur not only in Sertoli-lineage cells, but also in other types of cells that express *Nr5a1*. Early *Nr5a1* ablation might be involved in the induction of different profiles of gonadal marker expression between the two XY *Sox9-Cre-Nr5a1* cKO genotypes at E12.5‒E15.5, since *Nr5a1* expression differed between *Sox9-Cre;Nr5a1f.*^*/f*^ and *Sox9-Cre;Nr5a1f.*^*/−*^ gonads, at least at E12.5. This differential expression of *Nr5a1* between the two genotypes might be due to *Nr5a1* ablation in Sertoli cells and/or early *Nr5a1* ablation in all cells expressing *Nr5a1*. Sertoli cells from the gonads of both genotypes need to be sorted using FACS, and qRT-PCR analysis of *Nr5a1* is needed to address this issue. Early *Nr5a1* ablation via the *Sox9-Cre* system might also have induced a phenotype in XX *Sox9-Cre-Nr5a1* cKO mice.

The results of IHC double staining to detect SOX9 and FOXL2 showed that the decrease in the numbers of SOX9^+^ Sertoli cells was accompanied by a gradual increase in that of FOXL2^+^ cells in both XY *Sox9-Cre-Nr5a1* cKO models at E13.5–E14.5. Sertoli cell differentiation spatially spreads in a centre-to-pole pattern in XY gonads^19^, while FOXL2 expression begins at the anterior-dorsal side in XX gonads^19^. We detected SOX9^+^ and FOXL2^+^ cells in the central and pole-to-dorsal regions, respectively, in XY *Sox9-Cre-Nr5a1* cKO gonads, suggesting a blockage of Sertoli cell differentiation and the initiation of granulosa cell differentiation within the gonads. Testis cords were concomitantly irregular in XY *Sox9-Cre-Nr5a1* cKO gonads from E12.5. Since the formation and maintenance of testis cords require Sertoli cells^1,20^, the different severity of cord disruption between the two *Sox9-Cre-Nr5a1* cKO genotypes probably reflects differences in the sizes of Sertoli cell populations.

Although most AMH^+^ cells in XY control gonads were positive for NR5A1 (NR5A1^+^/AMH^+^), the gonads of the two XY *Sox9-Cre-Nr5a1* cKO models contained NR5A1^−^/AMH^+^ cells, indicating that AMH^+^ cells can differentiate in the absence of NR5A1. Despite disrupted NR5A1 binding to the *Amh* gene promoter, *Amh* transcription initiated in foetal testes produced *Amh* mRNA at levels sufficient for the induction of Müllerian duct regression, suggesting that NR5A1 is a quantitative regulator of *Amh* transcripts^21^. However, mutations of the SOX9-binding site in that study prevented *Amh* transcription, suggesting that SOX9 acts as an essential initiator of *Amh* transcription^21^. Furthermore, although Gata-binding protein 4 (GATA4) is also considered as an essential regulator of *Amh* transcription, *Amh* can be induced in Sertoli cells without GATA4 binding to the *Amh* promoter, thus leading to Müllerian duct regression^22^. Therefore, appropriate transcription of the *Amh* gene requires the cooperative action of several transcription factors, including NR5A1, SOX9, GATA4, Wilms tumour suppressor gene 1, and NR0B1 (also called Dax1) during testis development. Although the disruption of any one of these factors might alter interactions among them, some *Amh* might still be generated.

The development of the reproductive organs of both sexes is dependent on hormones secreted from the testis during a critical period of embryonic development^23,24^. In males, AMH induces the regression of the Müllerian ducts, and testosterone acts on the Wolffian ducts that develop into the epididymides, vas deferens, and seminal vesicles. In females that do not have AMH, the Müllerian ducts develop into the oviducts, uterus, and upper vagina, and the Wolffian ducts regress. The numbers of AMH^+^ cells and levels of *Amh* were obviously reduced at E12.5‒E14.5 in both XY *Sox9-Cre*-*Nr5a1* cKO models in the present study. Müllerian ducts had histologically ambiguous epithelial mesenchymal borders with TUNEL-positive apoptotic cells in XY control mice, whereas those in both XY *Sox9-Cre*-*Nr5a1* cKO models retained clear tubular structures without apoptotic cells, similar to those in XX control mice at E14.5 (Supplementary Fig. 6). These results indicate that insufficient AMH/*Amh* during the critical period induced persistent Müllerian ducts and the subsequent development of the uterus in XY *Sox9-Cre*-*Nr5a1* cKO mice.

The vas deferens and seminal vesicles were present in XY *Sox9-Cre;Nr5a1f.*^*/f*^ mice but not in XY *Sox9-Cre;Nr5a1f.*^*/−*^ mice. We did not measure testosterone levels, but they were probably the main determinant of Wolffian duct development in XY *Sox9-Cre;Nr5a1f.*^*/f*^ and XY *Sox9-Cre;Nr5a1f.*^*/−*^ gonads. Since foetal Leydig cells lack 17 beta-hydroxysteroid dehydrogenase (17βHSD), which converts androstenedione to testosterone during steroidogenesis, they co-operate with Sertoli cells that possess 17βHSD to produce testosterone^25^. The different testosterone levels might be due to the different numbers of Sertoli cells between the two cKO models.

Foetal Leydig cells also secrete insulin-like peptide 3 (INSL3), which influences testis descent^26^. XY *Sox9-Cre;Nr5a1f.*^*/f*^ gonads were located at the level of the bladder within the abdominal cavity, indicating insufficient INSL3.

Significantly fewer cells expressed the Leydig cell marker 3βHSD in the cKO models than in XY controls at E13.5, and qRT-PCR findings showed significantly lower expression of the Leydig cell marker *Cyp11a1*, in XY *Sox9-Cre*-*Nr5a1* cKO, than in XY control gonads at E12.5–E13.5. These results indicated that Leydig cell differentiation is also affected. As described above, *Nr5a1* could become ablated in cells expressing *Nr5a1* including Leydig-lineage cells at indifferent stages. Since foetal Leydig cell differentiation is regulated by paracrine factors secreted from Sertoli cells, including AMH and DHH^27^, foetal Leydig cell differentiation might be indirectly affected by the reduced numbers of Sertoli cells. However, subsequent Leydig cell fates greatly differed between gonads of the two XY *Sox9-Cre-Nr5a1* cKO models. The numbers of 3βHSD^+^ cells in XY *Sox9-Cre;Nr5a1f.*^*/f*^ recovered to levels equivalent to those in XY controls by E14.5, whereas those in XY *Sox9-Cre;Nr5a1f.*^*/−*^ became undetectable by E15.5. Further studies are needed to understand the mechanistic differences underlying these phenotypes.

The presence of DDX4^+^ cells and normal *Ddx4* levels in both XY *Sox9-Cre-Nr5a1* cKO models suggested that germ cells entered the gonads normally; therefore, their irregular distribution is probably due to testis cord disruption. The presence of STRA8^+^ cells in the gonads of both XY *Sox9-Cre-Nr5a1* cKO models indicates meiotic entry of germ cells. The expression of STRA8 in females is notably induced by retinoic acid (RA), which is synthesised in the mesonephros, whereas germ cells in males are protected from RA exposure by cytochrome P450 family 26 subfamily B member 1 (CYP26B1), an RA metabolising enzyme, which is produced by Sertoli cells and regulated by NR5A1 and SOX9^28,29^. The meiotic entry confirmed herein might have been caused by insufficient CYP26B1 expression due to the reduced numbers of Sertoli cells. Since the meiotic entry of germ cells spatially proceeds from the anteroposterior regions to the dorsoventral regions of the gonads along the diffusion flow of RA from the mesonephros into the gonad^19^, localisation of STRA8^+^ cells to the pole-to-dorsal regions indicated that male-to-female sex reversal occurred in somatic and germ cells at these regions in XY *Sox9-Cre-Nr5a1* cKO gonads.

In summary, our results indicated that both male and female programmes in XY *Sox9-Cre;Nr5a1f.*^*/f*^ gonads were ongoing at least until E15.5, whereas the male pathway was abolished by E15.5 in XY *Sox9-Cre;Nr5a1f.*^*/−*^ gonads, suggesting that the dissimilar developmental profiles resulted in different reproductive phenotypes between the two cKOs. To the best of our knowledge, we showed that the early loss of *Nr5a1* in XY mice can induce gonadal sex reversal, providing the first evidence supporting a critical role of NR5A1 in gonadal sex determination in mice in vivo.

## Methods

All methods were carried out in accordance with ARRIVE guidelines.

### Animals

All procedures were conducted in accordance with the National Institutes of Health Guide for the Care and Use of Research Animals, and were approved by the Ethical Committee for Animal Use of the Aichi Gakuin University.

*Sox9-Cre* knock-in mice^18^ were provided by the RIKEN BRC through the National Bio-Resource Project of AMED (Tsukuba, Japan). *Nr5a1f.*^*/f*^ mice on a mixed 129/C57BL6 genetic background were obtained from the Jackson laboratory (Bar Harbour, ME, USA). To produce a more uniform phenotype, *Nr5a1f.*^*/f*^ mice on a mixed genetic background were backcrossed onto the C57BL/6 background for at least 6 to 8 generations. *Nr5a1f.*^*/f*^ mice were crossed with the *Sox9-Cre* (C57BL/6) transgenic mouse line to generate *Sox9-Cre;Nr5a1f.*^*/*+^ offspring. These mice did not show an obvious phenotype and were backcrossed with *Nr5a1f.*^*/f*^ mice to obtain XY *Sox9-Cre;Nr5a1f.*^*/f*^ mice. Alternatively, *Sox9-Cre;Nr5a1*^+*/−*^ mice were crossed with *Nr5a1f.*^*/f*^ mice to generate *Sox9-Cre;Nr5a1f.*^*/−*^ mice. All mice had free access to water and standard rodent chow, and were housed under 12 h light/12 h dark cycles. The mice were genotyped by PCR using specific primer sets (Supplementary Fig. 7) and sequences (Supplementary Table 1). We defined noon on the day when a vaginal plug was identified as E0.5.

### Histology, IHC, and terminal deoxynucleotidyl transferase deoxy-UTP-nick end labelling (TUNEL) assay

Embryos obtained at E12.5, E13.5, E14.5, and E15.5 were fixed overnight in 4% paraformaldehyde at 4 °C. Postnatal animals aged 2–3 weeks were perfusion-fixed with 4% paraformaldehyde, then gonads were dissected and stored in the same fixative overnight at 4 °C. Embryos and postnatal gonads were deparaffinised and rehydrated for IHC and for histological analysis by staining with haematoxylin and eosin. Antigen retrieval was performed via microwave-boiling for 5 min in 0.01 M citrate buffer. Endogenous peroxidase activity was quenched by immersing sections in 3% (v/v) hydrogen peroxide in PBS for 15 min. After three washes in PBS, the sections were incubated with 5% (w/v) Block Ace (Dainippon Pharmaceutical Co., Ltd., Tokyo, Japan) at room temperature for 30 min, followed by primary antibodies at appropriate dilutions overnight at 4 °C. The primary antibodies were rabbit anti-NR5A1 (1:10,000)^9^, goat anti-AMH (1:5000, sc-6886; Santa Cruz Biotechnology, Inc., Dallas, TX, USA), rabbit anti-SOX9 (1:10,000, ab185230), rabbit anti-3βHSD (1:1000, ab65156), rabbit anti-STRA8 (1:1000, ab49602) (all from Abcam, Cambridge, UK), goat anti-FOXL2 (1:10,000; Novus Biologicals, Littleton, CO, USA), and mouse anti-DDX4 antibody (1:2000, PA1-111A; Thermo Fisher Scientific Inc., Rockford, IL, USA). The sections were washed with PBS containing 0.1% (v/v) Triton X, followed by either N-Histofine Simple Stain Mouse MAX PO (R) (Nichirei Biosciences, Tokyo, Japan) or N-Histofine Simple Stain Mouse MAX PO (M) (Nichirei). The peroxidase reaction was visualised using Vector NovaRED (Vector Laboratories, Burlingame, CA, USA). Sections were dehydrated and mounted with Mount-Quick (Daido Sangyo, Saitama, Japan). The sections were double-stained for IHC by co-incubating polyclonal rabbit antibodies with monoclonal mouse or polyclonal goat primary antibodies. Sections were then incubated with donkey anti-mouse, anti-goat, and anti-rabbit secondary antibodies conjugated with Alexa 488 and Alexa 555 for 2 h, then washed with PBS. Nuclei were counterstained with 4,6-diamidino-2-phenylindole (DAPI; 1:1000, Sigma-Aldrich, St. Louis, MO, USA), and mounted using Fluoromount (Diagnostic BioSystems, Pleasanton, CA, USA). Apoptotic cells in Müllerian and Wolffian ducts were analysed by staining DNA strand breaks by TUNEL assays using TdT In Situ Apoptosis Detection Kits (R&D Systems, Minneapolis, MN, USA) as per the manufacturer’s instructions.

Sections from at least three gonads in each genotype group at each time point were processed in parallel to reproducibly compare immunostaining on at least two separate occasions. Negative control tissue sections were incubated without primary antibodies. Digital images were acquired using a Keyence BIOREVO microscope (BZ-9000; Keyence, Osaka, Japan) and transferred to Photoshop CS (Adobe Systems, Mountain View, CA, USA) to generate figures. Contrast, brightness, and cropping in all images were adjusted. Investigators who were blinded to the assignments of gonads quantified IHC data derived from at least three gonads from three mice per genotype.

### qRT-PCR

Gonads were dissected from E12.5 and E13.5 embryos in PBS, and two gonads from each embryo were combined. Total RNA (500 ng) extracted from gonads using RNeasy Micro Kits (QIAGEN, Hilden, Germany) was reverse-transcribed using the QuantiTect Reverse Transcription Kits (QIAGEN) as described by the manufacturer. Complementary DNA was analysed in triplicate using at least three independent biological replicates by qRT-PCR using a StepOne Real-Time PCR System (Applied Biosystems, Foster City, CA, USA), with fluorescence monitoring using the TaqMan Fast Advanced Master Mix Product Insert (Applied Biosystems). Gene-specific primers and probes for *Nr5a1* (Mm00446826_ml), *Sox9* (Mm00448840_m1), *Amh* (Mm00431795_g1), *Dhh* (Mm00432820_g1), *Cyp11a1* (Mm00490735_m1), *Foxl2* (Mm00843544_s1), *Fst* (Mm00514982_m1), *Ddx4* (Mm00802445_m1), and *18S ribosomal RNA* control reagents (APL-4308329) were obtained from Applied Biosystems. The cycling conditions were as follows: 95° for 20 s followed by 40 cycles of 95 °C for 1 s and 60 °C for 20 s, then Ct values were averaged. The internal control was 18S ribosomal RNA, and results were analysed using the ΔΔCt method. The controls were XY *Nr5a1f.*^*/f*^, XY *Sox9-Cre;Nr5a1f.*^*/*+^ or XY *Nr5a1f.*^*/*+^ littermates (RQ = 1).

### Statistical analysis

Statistical differences were assessed using Student’s t-test. Differences were considered significant at P < 0.05. All data are reported as means ± SEM.

## Supplementary Information

Supplementary Information.

## Data Availability

All data generated or analysed during this study are included in this published article.
