# Supplementary material for: The conditional deletion of steroidogenic factor 1 (Nr5a1) in Sox9-Cre mice compromises testis differentiation
Source: Sci Rep. 2021 Feb 24;11:4486. doi: 10.1038/s41598-021-84095-y (PMC7904858; doi:10.1038/s41598-021-84095-y)
Supplement: Supplementary file 1 — Supplementary Information. [file 41598_2021_84095_MOESM1_ESM.pdf]

The conditional deletion of steroidogenic factor 1 (*Nr5a1*) in *Sox9-Cre* mice compromises testis differentiation

Yayoi Ikeda[1, *], Ayako Tagami[1], Mamiko Maekawa[1], and Akiko Nagai[1]

[1]Department of Anatomy, Aichi-Gakuin University School of Dentistry, Nagoya, Japan

***Corresponding Author:**
Yayoi Ikeda, DDS, PhD
Department of Anatomy, School of Dentistry, Aichi Gakuin University
1-100 Kusumoto-Cho, Chikusa-ku, Nagoya, Aichi 464-8650, Japan
Tel: +81-52-757-6756
Fax: +81-52-757-6755
E-mail: yayoi@dpc.agu.ac.jp

Supplementary Figure 1

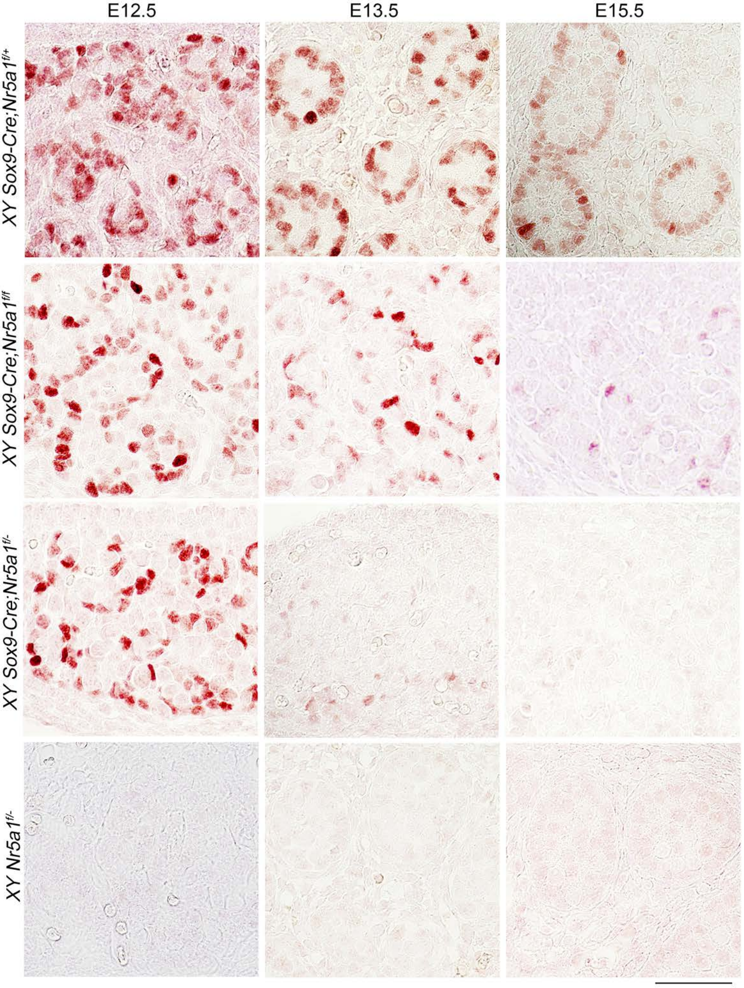

Supplementary Figure 1. Cre expression in XY *Sox9-Cre* gonads. IHC for Cre in gonads of XY *Sox9-Cre;Nr5a1*$^{f/+}$, XY *Sox9-Cre;Nr5a1*$^{f/f}$, XY *Sox9-Cre;Nr5a1*$^{f/-}$, and XY *Nr5a1*$^{f/-}$ mice at E12.5, E13.5, and E15.5 (left, middle, and right panels, respectively). Scale bar, 50 µm.

Supplementary Figure 2

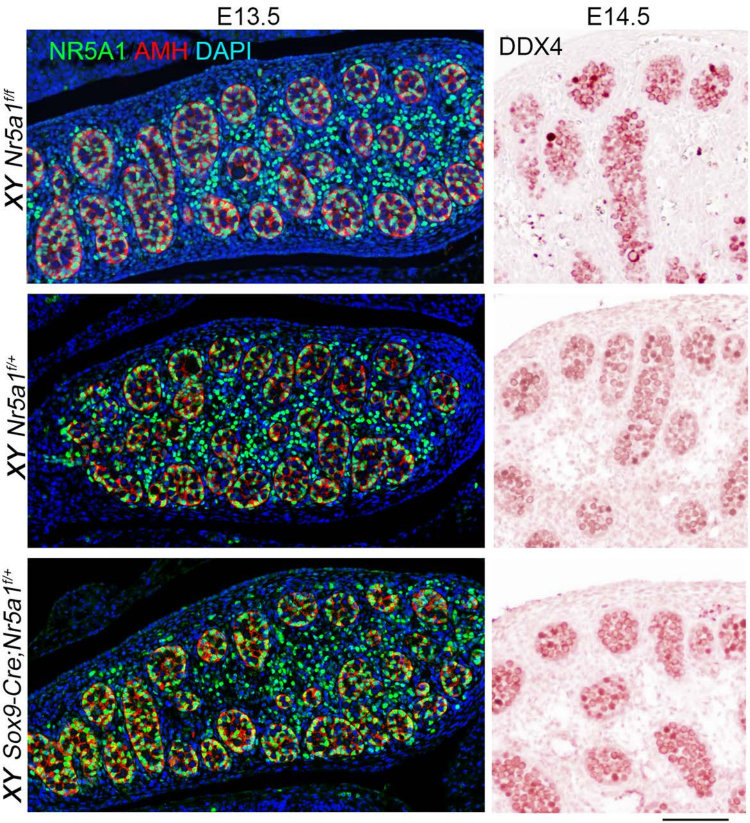

Supplementary Figure 2. IHC in three genotypes of XY control testes. Double IHC for NR5A1 and AMH at E13.5 and IHC for DDX4 at E14.5 in the testis of XY *Nr5a1*$^{f/f}$, XY *Nr5a1*$^{f/+}$, and XY *Sox9-Cre;Nr5a1*$^{f/+}$ mice. Scale bar,100 μm.

Supplementary Figure 3

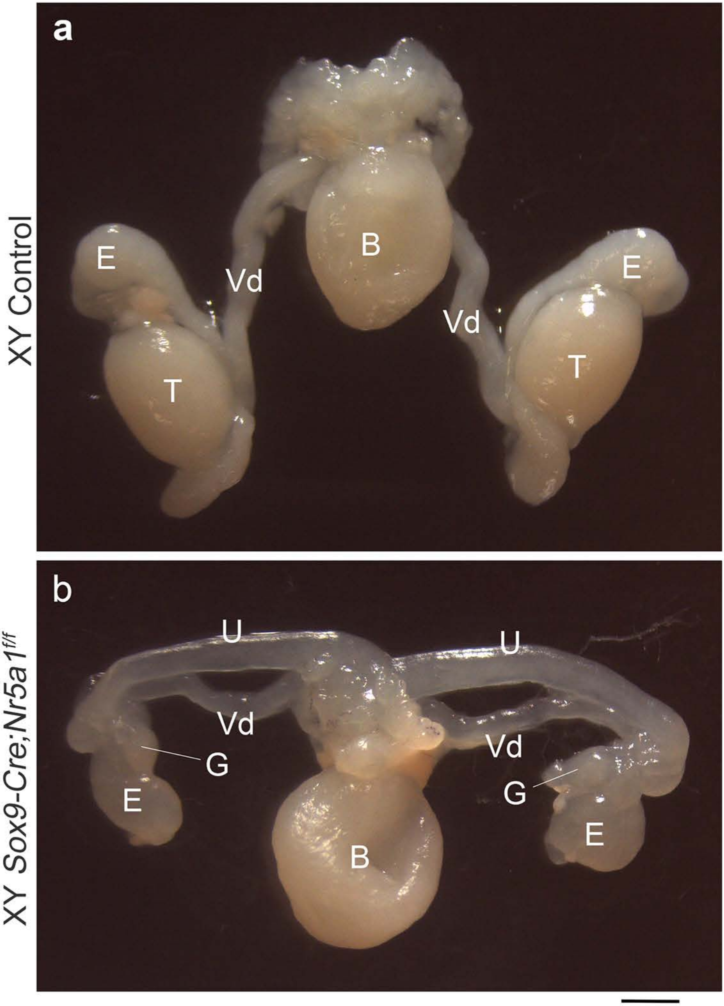

Supplementary Figure 3. Internal reproductive organs of XY *Sox9-Cre;Nr5a1f$^{f/f}$* mice. Stereomicroscopy images of internal reproductive organs in XY control (upper panel) and XY *Sox9-Cre;Nr5a1$^{f/f}$* (lower panel) mice at two week-old. B, bladder; E, epididymis; G, gonad; T, testis; U, uterus; Vd, vas deferens. Scale bar, 1 mm.

Supplementary Figure 4

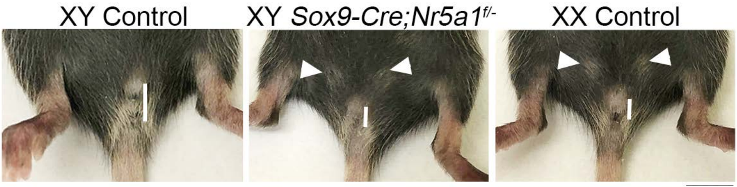

Supplementary Figure 4. External genitalia of XY *Sox9-Cre;Nr5a1*$^{f/-}$ mice. Images of external genitalia in XY control, XY *Sox9-Cre;Nr5a1*$^{f/-}$, and XX control mice (left, middle, and right panels, respectively) at two week-old. White bars and white arrowheads indicate anogenital distances and mammary teats, respectively. Scale bar, 1 cm.

Supplementary Figure 5

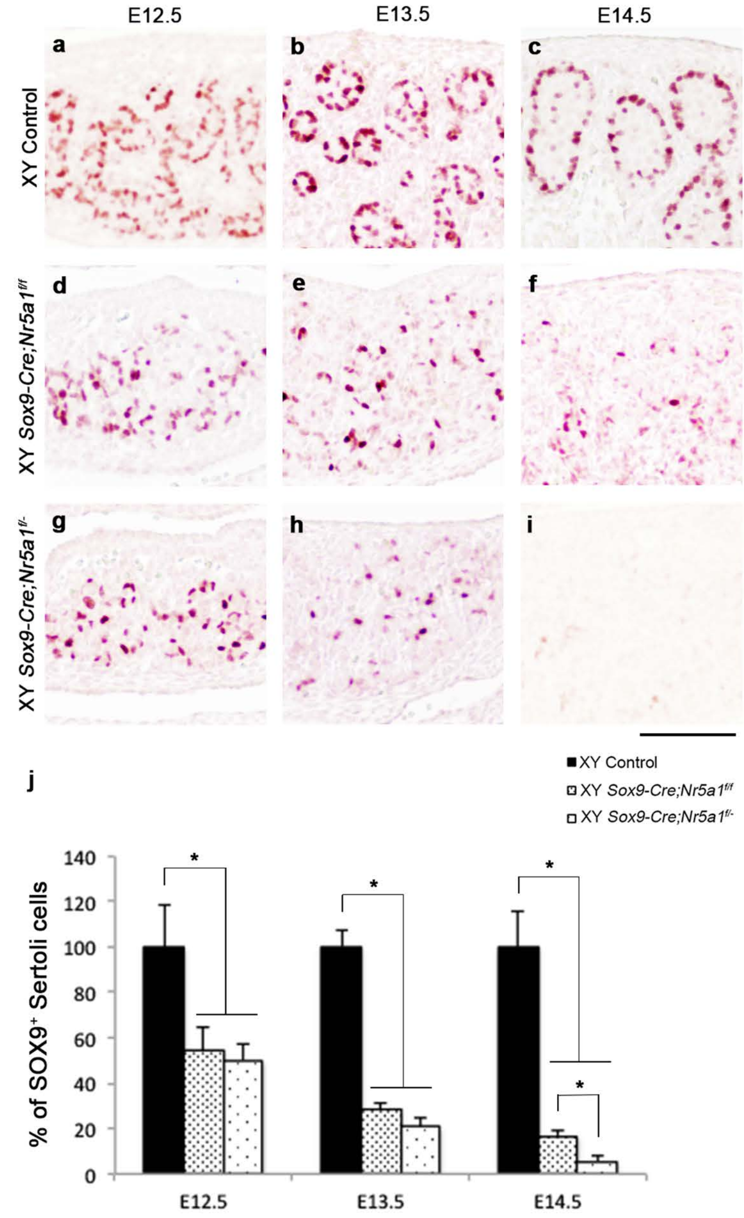

Supplementary Figure 5. SOX9 expression in XY *Sox9-Cre-Nr5a1* cKO gonads. IHC for SOX9 in gonads of XY control (a-c), XY *Sox9-Cre;Nr5a1*$^{f/f}$ (d-f), and XY *Sox9-Cre;Nr5a1*$^{f/-}$ (g-i) mice at E12.5, E13.5, and E14.5 (left, middle, and right panels, respectively). Scale bar, 100 μm. (j) Ratios (%) of SOX9^{+} cells. (Daily values for XY control were set at 100%). Data are shown as means ± SEM (n = 3). *P < 0.05 vs. XY control.

Supplementary Figure 6

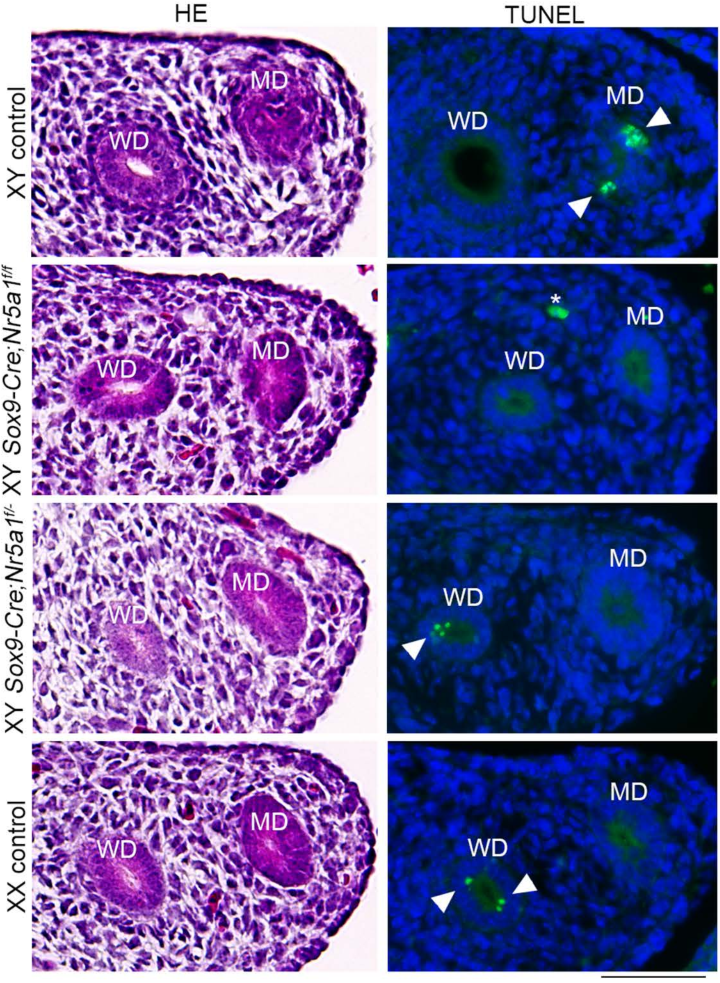

Supplementary Figure 6. Lack of Müllerian duct regression in XY *Sox9-Cre-Nr5a1* cKO mice. Haematoxylin and eosin (HE) staining and TUNEL assay in the mesonephroi in XY control, XY *Sox9-Cre;Nr5a1*$^{f/f}$, XY *Sox9-Cre;Nr5a1*$^{f/-}$, and XX control mice at E14.5. In TUNEL assay, nuclei are counterstained blue with DAPI. White arrowheads indicate TUNEL-positive apoptotic cells. White asterisk indicates nonspecific autofluorescence. MD, Müllerian duct; WD, Wolffian duct. Scale bar, 50 µm.

Supplementary Figure 7

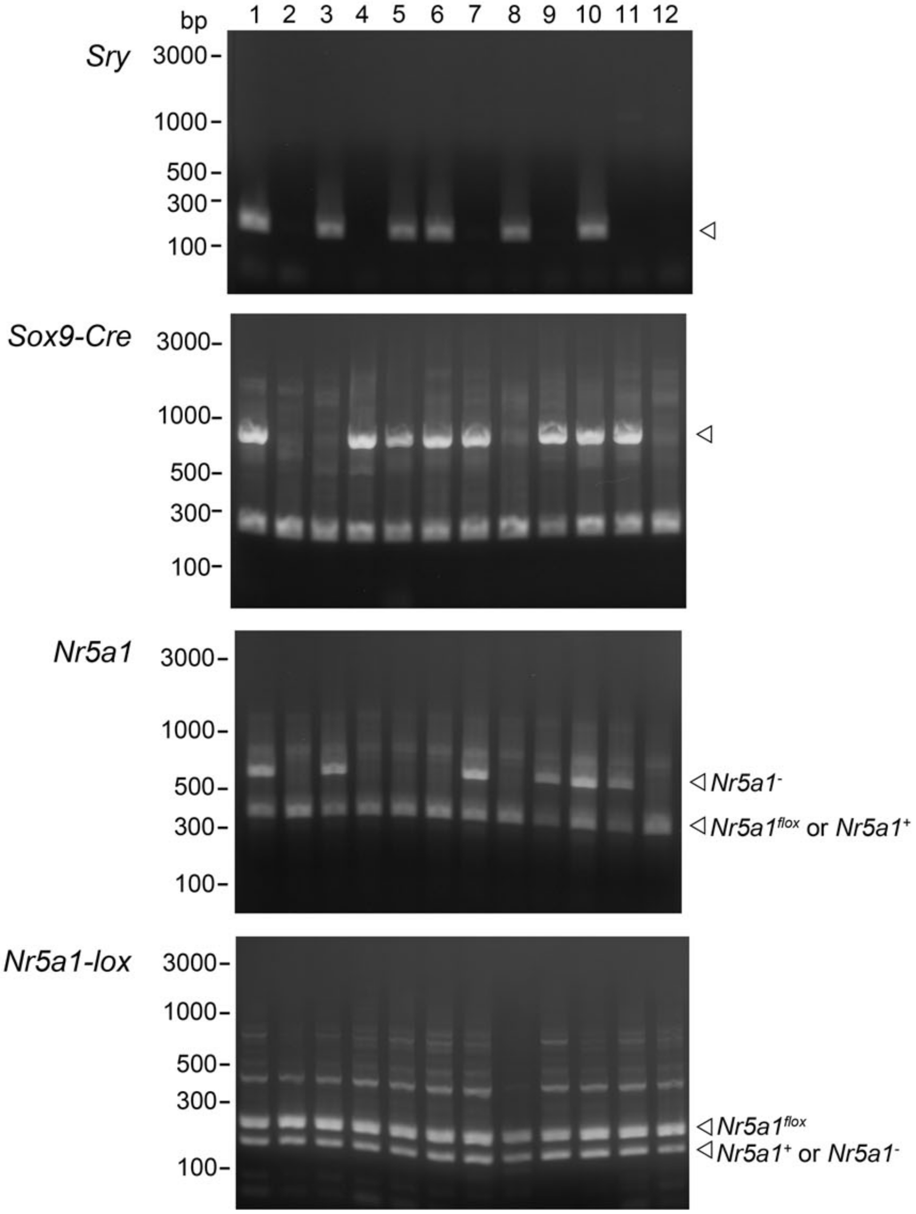

No	*Sry*	*Sox9*	*Nr5a1*	Genotype
1	+	*Cre*/+	*f*/-	cKO ♂
2	-	+/+	*f*/+	control ♀
3	+	+/+	*f*/-	hetero ♂
4	-	*Cre*/+	*f*/+	control ♀
5	+	*Cre*/+	*f*/+	control ♂
6	+	*Cre*/+	*f*/+	control ♂
7	-	*Cre*/+	*f*/-	cKO ♀
8	+	+/+	*f*/+	control ♂
9	-	*Cre*/+	*f*/-	cKO ♀
10	+	*Cre*/+	*f*/-	cKO ♂
11	-	*Cre*/+	*f*/-	cKO ♀
12	-	+/+	*f*/+	control ♀

Supplementary Figure 7. PCR genotyping and sexing of *Sox9-Cre;Nr5a1*$^{f/-}$ mice. A result of genotyping and sexing of 12 mice is shown.

Supplementary Table 1. Primers for genotyping

Gene	Primer name	Primer sequence
Sry	Sry-Forward	AAGCGCCCCATGAATGCATT
	Sry-Reverse	CGATGAGGCTGATATTTATA
Sox9-Cre	Sox9-Forward	CCAGATGGACCCACCAGTATCAG
	Sox9-Reverse	GGGACACTCTTGAACTAGGAGTAG
	CW-Cre2	TCCGGTTATTCAACTTGCACCATGC
Nr5a1	Nr5a1-Forward	ACAAGCATTACACGTGCACC
	Nr5a1-Reverse	TGACTAGCAACCACCTTGCC
	Neo Nr5a1	AGGTGAGATGACAGGAGATC
Nr5a1-lox	Nr5a1-lox-Forward	AGACAAGTGCACCCCATTTC
	Nr5a1-lox-Reverse	ACCATCACCAACCGCTAAAC
